# Algae biodiesel - a feasibility report

**DOI:** 10.1186/1752-153X-6-S1-S1

**Published:** 2012-04-23

**Authors:** Yihe Gao, Chapin Gregor, Yuanjie Liang, Dawei Tang, Caitlin Tweed

**Affiliations:** 1 University of Chicago, Chicago, 60637, USA; 2 American University, Washington DC, 20016, USA

## Abstract

**Background:**

Algae biofuels have been studied numerous times including the Aquatic Species program in 1978 in the U.S., smaller laboratory research projects and private programs.

**Results:**

Using Molina Grima 2003 and Department of Energy figures, captial costs and operating costs of the closed systems and open systems were estimated. Cost per gallon of conservative estimates yielded $1,292.05 and $114.94 for closed and open ponds respectively. Contingency scenarios were generated in which cost per gallon of closed system biofuels would reach $17.54 under the generous conditions of 60% yield, 50% reduction in the capital costs and 50% hexane recovery. Price per gallon of open system produced fuel could reach $1.94 under generous assumptions of 30% yield and $0.2/kg CO_2_.

**Conclusions:**

Current subsidies could allow biodiesel to be produced economically under the generous conditions specified by the model.

## Background

Due to concerns about high or unpredictable energy prices, the uncertain continued availability of fossil fuels, and the desire to derive energy from sources not under the control of hostile nations, the United States has long supported the production of biofuels through various incentive programs. Beginning with the passage of the Energy Tax Act in 1978, which provided a 100% gasoline tax exemption for alcohol fuel blends [1], the United States’ policy has been greatly in favor of incentivizing the expansion of the use of biofuels. There are several reasons that biofuels are even more viable now than at any time in the past several decades. First, oil prices are significantly higher now than they were in the past and are not likely to fall to those low levels again. Biofuels are always seen as a more attractive option whenever fuel prices rise. Therefore, research into biofuels could be more cost-effective now, in an age of higher gas prices.

Second, though clean energy and environmentalism were concerns in the nineties, they are much more prominent on the nation’s policy agenda in the present. Fears regarding global warming and related potential environmental catastrophes have made the government much more open to considering expensive policy options with positive environmental externalities. Since environmental concerns are being weighted with much more importance today, biofuels are much more attractive now, especially when created from a feedstock that avoids the environmental detriments of large-scale farming.

Third, energy independence is more important to the U.S. government today than it was back in the nineties. Now, with the wars in Iraq and Afghanistan, a loss of progress in the Arab-Israeli conflict, and increased fears of terrorism as a result of the September 11^th^ attacks, any energy policy that can make the United States self-sufficient, i.e. not having to rely on such an unstable region for fuel, will be much more popular. Since biofuel is entirely a domestic product, it fits these criteria quite well.

Finally, the current recession may be an important impetus to investment in projects like production facilities for new types of biofuel. Much has been made of the importance of “shovel-ready” projects such as public works improvements for combating the recession. Indeed, the American Recovery and Reinvestment Act of 2009 earmarks over $61 billion for energy generation, efficiency improvements, and general research, including $800 million for projects specifically related to biomass [2]. It is clear that the government is currently interested in programs like the development of biofuel production capabilities as a way to stimulate domestic investment as well as to improve fuel generation and efficiency.

Despite these benefits, however, the time is not necessarily right for just any type of biofuel. There are many types of biofuels currently being researched and produced. Ethanol, biodiesel, and other oil-based fuels exist that can be either used directly in vehicles or that can be used after engine modifications or in blends with petro-fuels. We are choosing to look at biodiesel for several reasons. First, biodiesel can be used directly in diesel engines, whereas ethanol must be mixed with regular gasoline in order to work in gasoline engines (except those specially modified for ethanol only). Second, biodiesel takes less energy to make than petrodiesel does, making its net energy produced higher, even though the outputs of petro- and biodiesel are similar. Biodiesel also has lower emission rates of certain pollutants, such as SO_X_, CO, and particulate matter. Biodiesel eliminates tailpipe emissions of SO_X_ completely [3]. Most importantly, biodiesel is renewable, and we can control its production levels and methods in a variety of ways to ensure the desired outcomes. As petroleum gets harder to extract from the earth and therefore more costly, biodiesels will remain as cost-efficient as the processes required to produce them, and these processes can change with new technology.

While diesel and gasoline engines are quite similar, the differences are important. Diesel fuel will self-ignite when pressurized in the cylinder, whereas gasoline needs a spark from a spark plug to combust. Diesel fuel also has more carbon atoms per molecule than gasoline, thus the energy density of diesel is greater than that of gasoline (Table 1). Diesel engines are relatively more efficient than gasoline engines as well, though they are required to work at higher temperatures, so some energy is lost to heat. In order to make a regular diesel engine run on biodiesel, no conversion is necessary. This goes for all blends, from B2 (2% biodiesel, 98% conventional diesel) all the way to B100 (100% biodiesel). One small concern is that in cold weather (temperatures below 30 degrees F) biodiesel viscosity will increase, blocking fuel lines. This problem can be solved by mixing in additives, such as a higher percentage of petrodiesel, or by installing heaters for the fuel lines.

**Table 1:** Average density and heating values of biodiesel and diesel fuels

Fuel	Density (g/cm3)	Heating value avg., BTU/gal	% difference vs. no. 2 diesel avg.
87 Octane gasoline	0.740	116,090	10.36%
Ethanol (E100)	0.789	84,530	34.53%
No. 2 diesel	0.850	129,500	-
Biodiesel (B100)	0.880	118,296	8.65%
B20 blend	0.856	127,259	1.73%
B2 blend	0.851	129,276	0.17%

Biodiesel is more environmentally friendly than petrodiesel in many respects. One thing that biodiesel improves over petrodiesel is lubrication ability. In petrodiesel, environmental regulations require reduced sulfur content, but this sulfur was needed to increase lubrication. Biodiesel, however, does not need sulfur for lubrication and therefore is better for the environment in that regard [4]. Biodiesel vehicles also have significantly lower emissions when compared to standard diesel vehicles (Table 2).

**Table 2:** Engine emission results, in % difference from no. 2 diesel [4]

Emission	B100	B20
Hydrocarbons	-52.4%	-19.0%
Carbon monoxide	-47.6%	-26.1%
Nitrous oxides	-10.0%	-3.7%
Carbon dioxide	+0.9%	+0.7%
Particulate matter	+9.9%	-2.8%

Biodiesel not only burns more cleanly, but it may also have the advantage of being cleaner in its production process, depending on how one produces it. Recycled vegetable oil, for example, is an extremely clean feedstock for biodiesel because it has already been produced and used for other things. Relative to the production processes for these oils, the conversion costs tend to be slim or even negligible by comparison. Other feedstocks, however, are not as clean to make, and some may even counter-productively use more resources and release more carbon than petroleum-based fuel.

**Figure 1:**
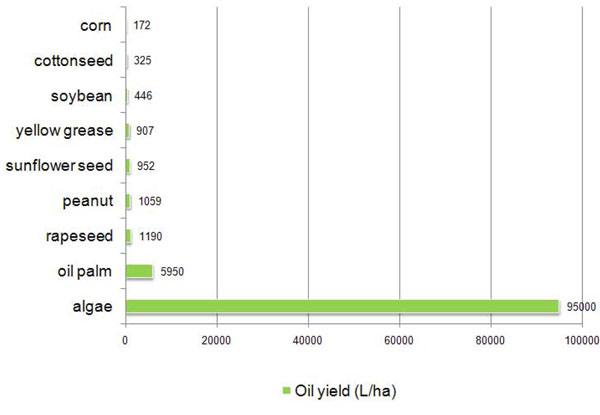
**Oil Yields of Feedstocks for Biofuel.** Numbers sourced from [20].

With all this in mind, we have chosen to consider not the broad category of biodiesels but rather the much more specific subcategory of algae fuel. There are many possible biofuel feedstocks, and there are several that are currently getting much more funding and attention than algae, but we chose algae because it seems to be the best hope for producing a fuel that might one day be cost competitive with petroleum fuels. The major feedstocks currently being used to produce oil for biodiesel are corn, soybean, rapeseed, yellow grease, and oil palm. Algae has the capability of yielding many times as much oil as the other feedstocks per unit of growing area, and that corn and soybeans are especially inefficient in this respect (Figure 1).

Algae biomass has the potential to grow yields far higher than any other feedstock currently being used. It has the possibility of a much higher energy yield per unit, so it can be much more efficient. However, little funding is being put into algae research currently. The main feedstocks being grown for biodiesel now are seeds, such as corn, soybean, rapeseed, and peanut. Yellow grease, which is used animal and vegetable fats (restaurant cooking oil and other fats) is also a very popular feedstock for biodiesel. As with other recycled oils, yellow grease biodiesel production is low cost, but it will not scale up to larger production, and thus production levels cannot be maintained as demand increases since it is recycled from other places where demand is not increasing.

The problem with the virgin oil feedstocks (not recycled) that are currently being put forward as good stocks for biodiesel production is that they are all farming-intensive. Plants such as corn and soy must be fertilized, irrigated, and maintained, and all those processes use up valuable resources, create soil erosion problems, and emit greenhouse gasses, as well as polluting in other ways (nitrogen runoff into water sources, for example). Some scientists claim that growing corn to make biofuels is more carbon-intensive than simply using petro-fuels instead [5], though this is widely disputed and depends on how one accounts for the costs of farming and fertilizer production. Another problem is that using food crops for biofuel increases the price of the food crop, which can lead to higher world food prices as we saw in 2007 [6]. While corn has gotten most of the bad press for being a very energy- and water-intensive crop to crow, all these farmed feedstocks have similar problems regarding energy and cost inputs and outputs.

Algae, on the other hand, can be easy to grow, and it does not require additional fertilizers or pesticides like many farmed crops do. It simply requires CO_2_ and sunlight to grow. It can be grown in grey water or wastewater, and in fact nitrogen-rich waste ponds are some of the better places to grow algae. It also can be grown on marginal land, so it does not take away from land used in farming for food. Algae gets its energy from the sun, so as with farmed crops, the energy output from algae biofuels does not require the direct input of other forms of chemical energy. Furthermore, the carbon released through biodiesel combustion was initially fixed from CO_2_ gas through photosynthesis. Thus, algae biodiesel is carbon neutral. In addition, algae have a much higher oil yield than any other feedstock currently being researched. Algae also have potential added benefits that have not yet been researched fully. One of these is the possibility of selling carbon credits or buying emitted CO_2_ from factories, further reducing overall greenhouse gas emissions. Another possible benefit could be selling the leftover nutrient-rich biomass from the algae to animal-raising farms as feed for livestock, as well as burning the leftover biomass for electricity to power the facility itself or to sell back to the grid.

Many scientists have recognized the problems with all these feedstocks for biofuel and have looked to genetically-engineered bacteria as the solution. Such bacteria, E. coli for example, can multiply much faster than any plant or algae, and they can be facilely engineered to produce precisely what the scientist wants. This solution seems like the ideal one for biofuel production. However, many have pointed out that bacteria merely reassemble current chemical energy sources at an energy cost, whereas algae harness solar energy and CO_2_ that would have otherwise been unused.

In the body of our paper, we will provide an overview of algae-related research up to the present time and then explain the scientific processes of producing biodiesel from algae. We will then conduct an economic feasibility analysis, taking into account public policy measures that could change the costs of production. Our conclusions will follow, showing the possible scenarios in which algae biodiesel could become cost-competitive as well as the scenarios in which it is not.

### Current research review

Research on algae fuel has been limited in the past, although the fluctuation in oil prices has ignited renewed interest in algal biodiesel for its high oil yield. Most research into the efficiency of algal-oil production continues to be done in the private sector, while government emphasis on algae research varies from country to country. For this paper, we will focus on one of the largest public funded program dedicated to algae research in the United States.

### Algae research in the public sector

In the United States, the earliest government funded research on algae began in 1978 under the Carter administration. It was known as the Aquatic Species Program (ASP) and was funded by the Department of Energy (DOE) under the Office of Fuels Development. The ASP was just one component of the larger Biofuel Program under the DOE that aims to develop alternative sources of energy domestically in the United States, and its report was completed in 1998 [7]. Prior to 1980, the ASP started out focusing on using algae to produce hydrogen, but the DOE gradually shifted its emphasis on technologies that could have a large-scale impact on national energy consumption after 1980 and therefore prompted the ASP to focus on algae’s ability to produce biodiesel. The ASP can be divided into two components of research – laboratory studies and outdoor studies. While the laboratory studies are involved with investigating algae’s composition and oil yield, outdoor studies are concerned about testing large-scale systems and analyzing the cost-efficiency. Both components of research were carried on concurrently during the eighteen years of the program and build on each other’s findings in the process. A summary of the research timeline of the ASP is located in Table 3.

**Table 3:** Timeline for the aquatic species program

	Lab studies	Outdoor studies
**1980-1985**	Collection & screening of algae	Wastewater treatment, small pond studies

**1985-1990**	Classification of algae biochemistry & physiology of lipid production	Large pond studies

**1990-1996**	Genetic engineering	Systems analysis & resource assessment

#### Laboratory studies on algae

Within the laboratory studies, research is generally broken down into three types of activities: 1) collection, screening and classification of algae, 2) biochemical and physiological studies on lipid production, and 3) molecular biology and genetic engineering. The logical order of the three activities is very important to laboratory studies. Scientists are required to first gather a substantial amount of information on algae through collection and classification. Then, once adequate information is gathered, research can focus on oil production through understanding the biochemistry and physiology of algae. A natural next step is therefore to use such knowledge to genetically manipulate the metabolism of algae to improve its oil production.

#### Collection and classification

Due to the large diversity of algae population, researchers were first interested in finding the algae that produced the most oil, has the fastest growth rate, and can grow under severe conditions such as extreme heat, pH, or salinity. Therefore, a large-scale operation took place from 1980 to 1987 dedicated to the gathering and screening of algae species. Collection first began in western Colorado, New Mexico, and Utah because it was believed that these harsh habitats will produce algae strains that can adapt to extreme environmental conditions. Subcontractors of the program were paid to collect algae strains from southeast regions such as Florida, Mississippi, and Alabama. Universities also joined the early collection efforts and collected large quantities of algae strains from various regions of the continental U.S. as well as Hawaii. By 1987, the collection consists of over 3,000 species of algae. The classification process began as soon as new strains enter the laboratories around the country and the resulting database was unprecedented in scale and serves a strong foundation for future research into algae.

#### Biochemistry and physiology

It quickly became apparent that no one single species was going to meet all the qualifications the ASP envisioned. Therefore, the research switched gear and concentrated on studying the biochemistry and physiology of oil production in the hope of learning how to improve the performance of existing organisms. Several major discoveries occurred in 1985, 1986, and 1988, when scientists discovered the so-called “lipid trigger” that lead to an increase of oil excretion in algae. “Lipid triggers” are chemical elements in algae nutrient that when removed, “starve” the algae such that a rapid buildup of oil droplets within the cells occurs. The first of these discoveries identified nitrogen as the trigger, with studies confirming observations that nitrogen depletion could lead to an increase level of oil present in many species of algae. The downside of the lipid trigger is its inhibitive effect on cell growth and therefore slows down the overall production rate. In 1986, the National Renewable Energy Laboratory (NREL) made another discovery while studying silicon depletion (Si-depletion) in diatoms (*Cyclotella cyptica*). They found that Si-depleted cells direct carbon more toward lipid production and less toward carbohydrate production. Hence, NREL researchers began to look for key enzymes in the lipid synthesis pathway to identify the critical factor for controlling oil production in algae.

#### Molecular biology and genetic engineering

**Figure 2:**
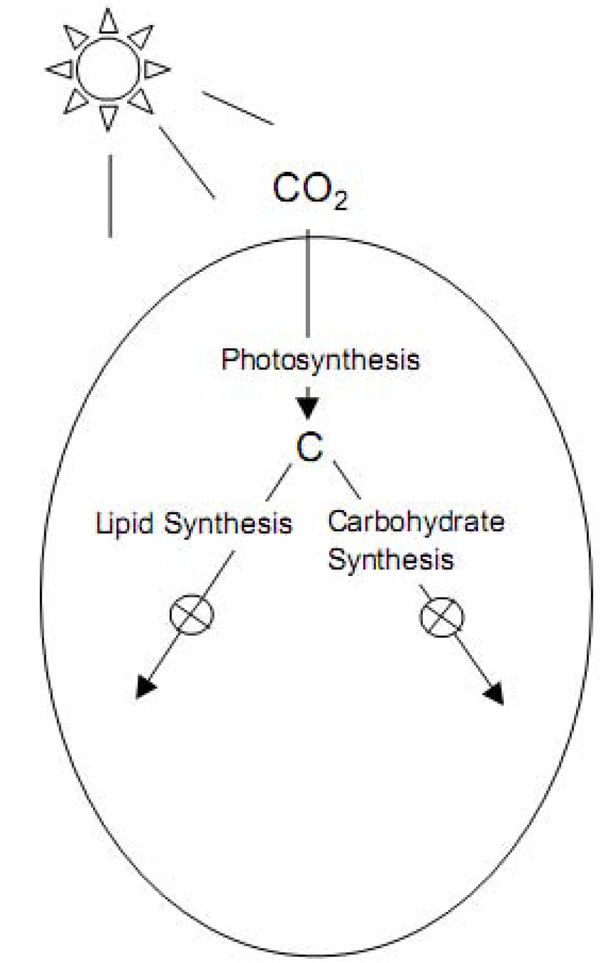
**“Carbon’s Metabolic Pathways” by NREL **[3]**.** Carbon goes through numerous metabolic pathways to synthesize compounds needed by the cells. Here’s a representation of two possible pathways. Diatoms store carbon in lipid form or carbohydrate form. The result of the NREL experiments suggests that it might be possible to alter which path the cells use for storage (e.g. more toward lipid synthesis, less to carbohydrate synthesis).

By 1988, researchers have successfully identified the enzyme *Acetyl CoA Carboxylase* (ACCase), which has shown positive correlation with lipid buildup during Si-depletion. These findings quickly prompted scientists to successfully clone the ACCase gene and to develop tools for expressing foreign genes in diatoms. In the 1990s, the ASP program accelerated rapidly and focused heavily on the genetic engineering front. At around the same time, another line of research that focused on the carbon metabolic pathway also yield a substantial discovery (Figure 2). Instead of focusing on the lipid synthesis, scientists identified key enzymes involved in the synthesis of carbohydrate and ways to disable them, thus diverting carbon to flow down the lipid synthesis pathway. However, the benefits of these findings have yet outweighed the loss from inhibitive growth rate due to depleted cells. Molecular research still needs to balance the efficiency of lipid production with algae growth, because those are two essential criteria for a viable algae farm environment.

At the current rate, laboratory studies will be a long-term effort, even after demonstration of potential for improving lipid production in algae and successful genetic reproduction. Many other factors are still required for algal mass culture, and some of them cannot be demonstrated in laboratories. Factors such as competitiveness, predation resistance, and harvestability are only feasible in outdoor testing. A strictly laboratory-based R&D program may lose touch with the realities of the eventual applications, thus, outdoor research must be carried out in parallel with laboratories studies

#### Outdoor studies on algae

Within the outdoor studies, research is also broken down into three categories: 1) wastewater treatment, 2) pond studies 3) system analysis and resource assessment.

Similar to the laboratory studies, the order of the above categories help describe the trajectory of algae research in the outdoor environment. Wastewater treatment was in development well before 1980 due to its essential role in urban planning. Since many early practices rely on expensive and sometimes environment-unfriendly chemicals or in using considerable amounts of energy, algae were used to provide a cheap and efficient alternative to those old practices. While waste streams serve as desired breeding grounds for algae populations, another gain from algae-based wastewater treatment is the end product – algae biomass, which can be used as a biofuel feedstock. Most past experiments evaluate a combined wastewater treatment/fuel production system based on microalgae. When the Aquatic Species Program took on this track, the emphasis had moved from algae based wastewater treatment to dedicated algae farming.

##### Algae pond studies

Research into algae for mass-production has mostly focused on microalgae, the reasons being that microalgae have a more simplistic structure, a fast growth rate, and high oil content. From 1980 to 1987, the ASP funded two parallel efforts to develop large scale mass culture systems of microalgae. One was called “High Rate Pond” (HRP) design, developed at UC Berkley. The other was called “Algae Raceway Production System”, developed by the University of Hawaii. The HRP design was ultimately selected by the ASP for the scale-up procedure and the “Outdoor Test Facility” (OTF) was constructed at an abandoned water treatment plant in Roswell, New Mexico. Between 1988 and 1990, the 1,000 meter pound achieved over 90% utilization of CO_2_. Best results were obtained using native algae species, which naturally had the fastest growth rate in their native climate. The OTF also demonstrated production of increased quantity of algae oil using both nitrogen and silicon depletion strategies from lab studies. The overall productivity was much lower than initially expected due to cold temperature days at the test site. The facility was closed down in 1990 and serve as a proof-of-concept for large scale open pound operation. Other outdoor projects were also funded over the course of the program including a subcontracted project with the Solar Energy Research Institute in Fairfield, California, which will be discussed more extensively in a subsequent section, and a three-year project in Israel. In the late 1980s, the Georgia Institute of Technology successfully developed the Algal Pond Model, a computer modeling tool for predicting performance of outdoor pond systems.

##### Resource assessment

Resource Assessment aims to address the issue of resource availability and utilization: Where can such technology achieve the maximum potential? Various resource analyses indicated significant potential of land, water, and CO_2_ resources in the southwestern United States, which provided the most suitable location for large-scale algae farming. For example, one study conducted by NREL concluded that there is a potential for production of several quads (10^15^ Btu) of biodiesel fuels in the southwestern U.S. alone. However, this does not take into consideration of the spread of resources in this vast region. It will be difficult to find many locations where all the resources for microalgae cultivation mentioned above are readily available. Furthermore, most coal-fired power plants in the United States are located in the north, or in otherwise unfavorable climates, so only a small fraction of power plant CO_2_ resources would be available to microalgae systems. Therefore, the resource potential estimated by some of the studies must be significantly discounted.

##### System analysis

Engineering design and cost analyses, together known as systems analysis, aim to address important questions relating to cost-efficiency of microalgae system: how much impact can algae technology have on current state of energy consumption? This is required both by the mission of DOE as well as the inherent need to justify budgetary decisions. The study analyses generally supported the view that microalgae biomass production could be performed at sufficiently low cost as to plausibly become a renewable energy source. The system analyses studies conducted under the ASP are much more accurate compared to earlier studies in the 1970s. Two systems – opened pond and closed photobioreactor – are the heart of the ASP program. One the one hand, the closed algae system provides better control over environmental conditions and biological contaminants, and higher productivities and harvesting rates. But the cost is extremely high and unfeasible at the current rate. On the other hand, large open pond systems are much more affordable, but at the same time due to hydraulic and CO_2_ supply limitation, productivity rate is still relatively low.

However, the most important issue involved in these engineering design and cost analyses are not the cost estimates, but the biological assumptions on which such designs are based. There has been a dramatic increase in projected productivities (from 50mt/ha/y in 1977 to 300mt/ha/y in 1993). This large increase is partially due to significant advances in scientific measurement, but also driven by clear necessity. Therefore, the main problem facing R&D involves less with engineering design, but more on dealing with microalgae cultivation, species control, and overall lipid harvest productivity. Future research will focus on these biological issues in the quest for low-cost production processes.

The total cost of the Aquatic Species Program is $25.05 million over a twenty-year period, compared to the total spending under the Biofuel Program ($458 million over the same period). In 1995, the DOE eliminated funding for algae research within the Biofuel Program. Under pressure to reduce budgets, the Department chose a strategy of more narrowly focusing its limited resources in one or two key areas, the largest of these is the development of bioethnaol.

### Algae research in the private sector

At present, most companies in the private sector are early stage start-ups that involved heavily in R&D rather than commercialization, many of them younger than five years old. To this day, none has launched a successful full-scale commercialization of biodiesel from algae. Most of the challenges facing these private companies are finance related, since a substantial amount of resources is required to set up the algae farming operation and venture capital is relatively scarce in this particular segment in comparison to other green initiatives. At the same time, many private companies’ R&D results produced innovative concepts and approach to biodiesel production. Unfortunately, we were not able to access most of the private research conducted within these companies. Nonetheless, we can look a few promising firms and their unique approach to algae commercialization, which is publicly available.

A few private firms have attracted media attention with their recent success in raising funding. Companies such as Massachusetts’s **Greenfuel Technologies Corporation** and California based **Solazyme** all utilized special closed systems. Greenfuel Tech builds algae bioreactor systems, which not only directly feeds recycled CO_2_ to the algae but also carefully control the algae’s intake of sunlight and nutrients. Solazyme, on the other hand specializes in using synthetic biology and genetic engineering to tweak algal strains for better biofuel yields. The company grows its algae in fermentation tanks without sunlight by feeding it sugar; both firms have already struck deals with biodiesel firms for the next stage of commercial expansion. Another California based firm called **LiveFuels** looks to continue the Aquatic Species Program’s research in using open-pond algae systems. The firm is trying to develop green crude to be integrated directly into the nation’s existing refinery infrastructure. Similarly, **Solix Biofuels**, a Colorado based company is also working on a biocrude, but using a closed-tank bioreactor set-up. The company has said that construction will begin shortly on its first, large-scale bioreactor at the nearby Belgian Brewery, where CO_2_ waste produced during the beer-making process will be used to feed the algae. Companies such as Seattle based **Inventure Chemical** and Israel based **Seambiotic** have teamed up to construct pilot commercial plants to produce algae for specific commercial applications. The combined effort will utilize high-yield oil-rich algae strains that Seambiotic has developed and grown in its open pound system coupled with Inventure’s patent-pending conversion processes to produce ethanol, biodiesel and other value-added chemicals. A new start-up **Aquaflow Binomics** from New Zealand is focusing on harvesting wild algae that can be grown in wastewater and local city waste streams, which doesn’t require extra land or feedstocks. The company has been working with Boeing on algae-to-bio-based jet fuel since last year.

### Algae production

#### Process overview

Since the large DOE funded Aquatic Species Program was halted in the 1996, however, much of the publicly funded research into algae biodiesels have taken place on a laboratory scale. The methods most commonly used in the literature, therefore, are associated with this small scale environment, and do not necessarily provide viable options for algae production when scaled up. Private ventures have no doubt furthered the research in this area, but since we do not have access to these documents, the following report on the current process by which algae is farmed and processed into biodiesel is extrapolated based upon these publicly available documents.

It is perhaps worthy of note that algae have been grown and harvested for a variety of reasons ranging from the production of algae as feed for zooplankton to the production of medically significant compounds or β-carotene. Although these processes often require different specifications for algal growth than those required for viable algal biodiesel production, it is also important to note that such large-scale algal farming efforts of the past can be used as roadmaps for the growing process, which may be of use for this particular endeavour.

**Figure 3:**
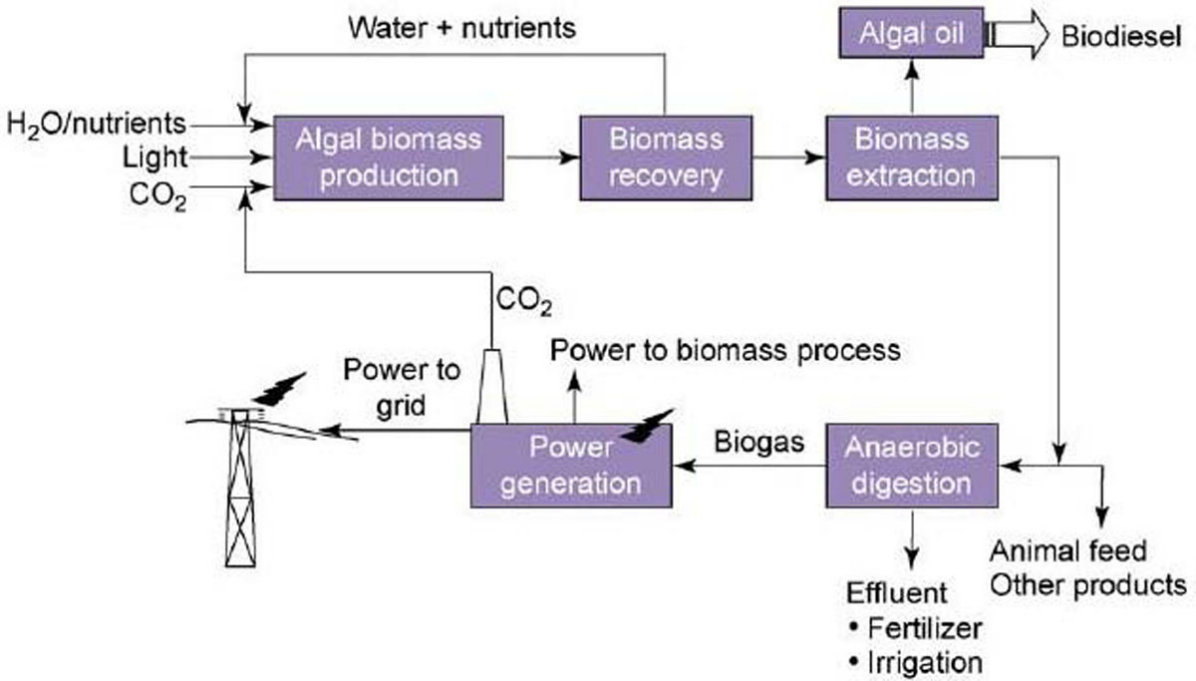
**Summary of the integrated process of algal biodiesel production **[10]**.** Water, inorganic nutrients, light, and CO2 are provided to the algal cultures in the algal 20 biomass production stage. Biomass is then separated from the water and nutrients in the biomass recovery stage, as the latter are recycled back into the algal cultures. The biomass then undergoes extraction to remove its lipid content. This lipid content is converted into biodiesel. Spent biomass can be used as animal feed or digested anaerobicly to generate gas for electricity while recycling CO2 emissions back into the biomass production stage.

It should also be noted that although several firms such as the New Zealand company, Aquaflow, have proposed to harvest wild algae, thereby bypassing the farming step of the process, most companies have proposed a process that involves the integration of growing, harvesting, and conversion of biomass into biodiesel. Water, nutrients, organic solvents, and carbon may be recycled through this process. Thus, there may be tangible advantages achieved through vertical integration of the algae biodiesel production process, which may reduce the amount of waste produced by the production process and reduce the costs from inputs. An overview of such a system is shown in Figure 3.

In this subsection we will provide an overview of the main steps of the biodiesel production process: choosing the species of algae, growing considerations, algae farm designs, processes for algal biomass recovery, extraction and conversion techniques, and current and future directions for research in this area to improve efficiency or productivity of the process.

#### Species of algae

Many species of algae have been researched with the intention of using these species as a potential feedstock for biodiesel. Of these species, *Botryococcus braunii*, has appeared in the literature as a laboratory favourite, although has not been commercially cultivated on a industrial scale. Table 4 lists some species of algae and their associated lipid content, but the table is not intended by any means to be comprehensive. In addition to the four species provided, there was also been a certain amount of interest in other algal species such as *Scenedesmus dimorphus*, *Euglena gracilis*, *Tetraselmis chui*, various *Spirulina* species, and many others that have been profiled as part of the ASP.

**Table 4:** Species of algae included lipid content and current cultivation [22-24]

Species	Lipid content (% dry weight)	Native habitat/current use	Advantages/problems
Botryococcus braunii	20-42% varying widely by strain	widespread in brackish lakes, reservoirs, ponds	slow to grow, not used for industrial production

Neochloris oleoabundans	23-40%		little known

Nannochloropsis salina	37%	already used extensively for zooplankton cultures	

Dunaliella tertiolecta	37%	has been cultivated to produce β-carotene	fast growing, high CO_2_ sequestration rate

### Production of algal biomass

#### Open ponds

**Figure 4:**
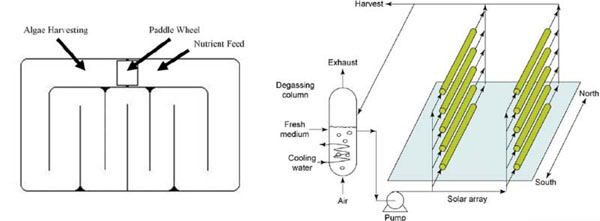
**“Raceway” Open Pond from Campbell **[21]**, Tubular Photobioreactor from Christi **[10]**.** The open pond design pictured represents a simplified and fairly common design for an open pond system. The photobioreactor system shows one possible flowpath by which harvesting, degassing, and nutrient replenishment might occur in a closed system.

The concept of an open pond system is relatively self-explanatory. The design requires for the carbonation of large pools (lagoons) of medium. In this regard, the name “open pond” may be misleading as the pond may be at least partially covered to maintain high enough CO_2_ concentrations. A 1987 report by the Solar Energy Research Institute [8] mentions several factors affecting the overall productivity of open pond systems including, but not limited to water resources, carbonation systems, mixing systems and harvesting systems. A basic design of an open pond can be found in Figure 4.

##### Water resources

With respect to water resources, much as been made about the ability to farm algae in salty or polluted waters, as it is common to see algae growing in polluted streams or ponds outside of an agricultural setting. With regard to open pond farming, however, since there is a relatively high cost associated with carbonation, the consideration of the properties of water can have an effect on the system costs. It was reported that in many cases hard water would require the addition of sodium carbonate, lime, or both depending upon the Ca and Mg content. The goal of selection was to minimize the dissolved CO_2_ levels at low pH while maximizing CO_2_ levels at the highest pH, where the former limit is set by the algae’s ability to tolerate increasingly alkaline growth media, and the latter limit is set by solubility constraints that result in outgassing. Obviously selection criteria are quite dependent upon the species of algae chosen.

##### Carbonation

According to the Weissman report, carbon is also among the most expensive inputs for an open pond system, its importance, however, should not be overlooked. A 1985 study by Chirac, et al. found that air that was enriched 1% CO_2_ lead to a 3.5x increase in the mean doubling time of growth of *B. braunii* as well as a 5-fold increase in its hydrocarbon production. This effect was not observed when bicarbonate was merely added to the growth medium [9]. As mentioned before, one possible solution is to cover the pond holding a high concentration of CO_2_ at the surface, thereby allowing the gas to passively diffuse into solution. Even so, the desorption of oxygen and nitrogen gases under the cover prevents us from having a high percent of coverage. Alternatively, it is also possible to inject CO_2_ from shallow stumps below the surface of the growth medium.

Once possibility for reducing carbon costs into the system is carbon recycling. The Weissman report estimates that in an open pond 60% of the algal biomass will be lipid, and only 90% of this biomass will be harvested. The remainder of the carbon-products will degrade either into gaseous products in the form of methane or CO_2_, or settle to become sludge or dissolve in the lagoon water. The gaseous products can be recollected and combusted to create a 35% CO_2_ mixture that can then be reinjected into the ponds. Christi further adds that surplus electricity generated by the combustion process can be sold to the grid.

##### Mixing systems

Constant agitation is also necessary in order to keep the cells in suspension, to disperse nutrients and prevent thermal stratification. The authors focused on two major means of mixing: the use of either paddle wheels or airlift mixing systems. Although the former was associated with greater initial capital costs, it was determined that there was insufficient data to properly assess the overall costs of the latter.

##### Other factors

Other factors important to algal growth, but not considered extensively in the Weissman report, include light intensity, temperature control, and the costs resulting from contamination, which open systems are relatively more vulnerable to, as opposed to closed systems.

#### Photobioreactors

There are different types of photobioreactor types available, of which the tubular variety is among the most commonly described in the literature, primary because this type of reactor has been used on a small scale for numerous laboratory assays. Figure 4 presents a diagram of such a design. Nevertheless, there is some doubt as to how well this system would work on an industrial scale. Christi [10] reports that this design calls for a solar collector consisting of an array of tubes containing the cell suspension, each 0.1m in diameter. During daylight hours, microalgae broth must be circulated throughout the system, and a high turbulence flow must be maintained at all times to prevent biomass sedimentation.

Algal growth is sensitive to levels of dissolved gases such as oxygen and carbon dioxide. Concentrations of oxygen much higher than air saturation values will inhibit photosynthesis, and at very high levels in combination with sunlight, could potentially damage the cells. Furthermore, algal growth is pH sensitive, an important consideration since the process of photosynthesis will naturally cause the tube pH to rise as greater amounts of dissolved CO_2_ are removed from solution as oxygen is introduced. Unlike in the open system, since the medium is enclosed within tubes, it is impossible for the gas to escape under ordinary circumstances. It is therefore impossible to run the tube reactors continuously, as the tubes must be periodically emptied for aeration and degassing.

Christi further reports that the sensitivity of the cells toward temperature often requires the introduction of cooling systems. Since the optimal growth temperature for the cells is 20-30°C, especially during daylight hours when constant exposure to sunlight heats the broth and could potentially damage the cells, cooling is essential. A heat exchanger or, in drier environments, evaporative cooling from water sprayed on the tubes, was deemed sufficient.

In a 1998 study by Sanchez Miron the comparative performance of several photobioreactor designs were reported for the culture of the microalga *Phaeodactylum tricorntum*. The microalgae were cultivated for the production of eicosapentaenoic acid, a potential treatment for certain cancers and heart disease reported in 1996. The report also includes a mention that a commercial horizontal tubular bioreactor facility in Cartagena, Spain was abandoned by its owner, Photobioreactors Ltd., after it failed to perform [11]. In general, photobioreactors appear to require a considerably larger compared with open pond systems.

#### Harvesting biomass

According to Weissman 1987, the standard protocol for the harvesting of algae from a dilute suspension of 0.05-0.1% consists of a concentration to reduce the volume of the sample by 20-50 fold followed by centrifugation, which, in turn reduces the remaining sample volume by 5-10 fold. Due to the near prohibitively high capital costs associated with centrifuges, however, it is necessary to examine other methods. A one-step separation of algae using an inclined or vibrating screen is also possible provided that packing of biomass on the screen continues to allow a high flow rate to be maintained. This latter process would allow for the effluents to be returned to the pond, but in this case, the harvesting process must not only remove the desired biomass, but also all potential contaminants. Other possible devices are dissolved gas floatation units, microstrainers, belt filters, and settling ponds. Of these devices dissolved gas floatation units had the highest capital cost (25 million 1987 USD/million gal of suspension/day), followed by belt filters (0.12), microstrainers (0.09), and settling ponds (0.05) although these latter methods are had costs of the same order of magnitude as of 1987. Any requirement to pre-treat the suspension prior to harvesting, however, will increase costs substantially, so as to trump any of the differences in capital costs associated with the price differences between these devices.

### Generating biodiesel

**Figure 5:**
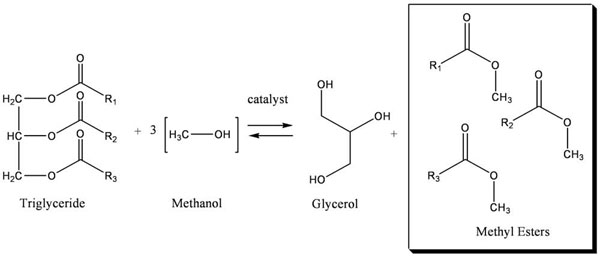
**The Transesterification Reaction.** During transesterification triglycerides obtained from biological products are processed with a three times excess of alcohol to generate glycerol and alkyl esters. Biodiesel consists largely of these alkyl esters; in the U.S. these must be monoalkyl esters.

Once the biomass has been dried two processes must occur in order to create biodiesel: the lipids must be extracted from the biomass and they must undergo a transesterification reaction (Figure 5). Although successful protocols have been established for these processes in the laboratory, it remains that these laboratory techniques are not particularly successful on an industrial scale with regard to algal biodiesel, although several reaction techniques have been used commercially for the transesterification of tallow and soybean.

The current technique for algal biodiesel production obtains lipids from biomass by means of standard grinding or sonication of the algal cells in order to lyse them, followed by extraction using organic solvents. Although such methods are common for harvesting biochemical products in the laboratory, the industrial scale equivalent usually requires the use to batch reactors, in which sonication and extraction take place in large vats. The harvested lipids are then reacted in a similar batch method using a dissolved or liquid catalyst and alcohol for the transesterification reaction. Generally, a standard reaction consists of methanol in a 6:1 molar ratio with oil input, 1:100 molar ratio of NaOH to oil input, and a 1:1 volume ratio of organic solvent to methanol [12].

However, there are several problems with batch reactor processes. The batch method does not allow for the lipids to be processed continually, as the vats must be emptied and refilled. The process has a requirement for a large amount of flammable organic solvents to be used, which could also pose some danger to workers. The use of the liquid catalyst also poses the problem that at the reaction’s end the catalyst is mixed with the products, and must be separated. It is for these reasons that batch reactors do not appear to be heavily used on an industrial scale, although they are heavily used in the laboratory. There are several references to commercial transesterification plants using a continuous flow system; however, there is a notable lack of information on the specifics of how these systems operate.

Some information is presented by Ben Wen, an investigator at United Environment & Energy (UEE), who is working on an improved continuous flow tubular reactor [13]. This is filled with a solid catalyst that does not leave the reactor. Oils are flowed through the reactor, undergoing transesterification as they pass the solid catalyst. Biodiesel can be generated at a greater rate using such a design since the tube does not need to be emptied and refilled. Furthermore, since the design of the tubular reactor is not vat-like, it can be smaller and, therefore, easier to transport. The great cost reduction within this system, however, is the elimination of the need to separate product and catalyst following the reaction. Although this design is currently in Phase I and being operated only on a small scale, with algal oil samples provided by outside producers, UEE reports that the design demonstrates greater scalability compared with traditional reaction processes and has partnered with other firms to design a complete algae biodiesel production process from algal growth to extraction and transesterification. It is highly likely that similar systems are utilized in existing plants.

### Current and future research directions

To date, publications in the scientific literature has indicated that much of the research into algal biofuels has focused on the treatment of algae and the optimization of its growth conditions varying factors such as nutrients in the growth medium, light, and gas content. Most of these optimization scenarios are designed to increase the algae productivity by increasing the algal growth rate or the lipid content. They take advantage of a highly controlled laboratory environment, but as one of the major obstacles facing the farming of algae in more cost effective open ponds is the threat of contamination, it is doubtful whether such research would be of direct usefulness to industrial production of algae. Other groups have characterized the different lipid compounds produced by various species or strains of algae, allowing for speculation on the characteristics of the products produced from these lipids.

Since the late 1980’s and early 1990’s, papers on algae farming have discussed the future use of genetic engineering as a means to greater productivity, whether of EPA or of lipids. Due to the great difficulty in genetic engineering of algae, however, this field is still in its infancy and little progress is evident from the literature.

Some inquiries have focused more on either the chemical features of the process or the industrial design aspect. We have already mentioned UEE in this regard, which in addition to reactor design, is attempting to optimize the form of algal biodiesel produced for performance in a standard diesel engine by reducing the biofuel’s sensitivity to oxidation and increasing its chemical stability. Research along these lines is rarer to find in journals, thus we speculate that most of this research is funded as part of private ventures. As a result, apart from press releases, such as those mentioned in the previous section, we do not have an estimate as to the extent that research has made inroads into these subjects.

## Methods, results and discussion

### Economic feasibility assessment

As noted before, there is very little publicly available research into algae farming. What little is available, however, is sufficient to form the basis of an economic analysis of algae farming for the purpose of producing biodiesel for both an open and closed system. For the closed system, we use a 2003 paper to form the basis of our evaluation, and a 1987 U.S. Department of Energy engineering report for the open system. For the analysis of the transesterification process, we reference several different sources. For the annualized capital costs of these systems, we discount them over ten years with a discount rate of 7%.

Molina Grima’s 2003 paper [14] estimates the cost for a closed bioreactor system based off direct experience and vender quotes. This system would produce 26.2 tons of algae biomass per year for the purpose of extracting a separate algae product, EPA. However, their process also produces oil as an intermediate product. Molina Grima assumes a 10% oil yield, leading to production of 2620 tons of oil a year, or about 787 gallons of oil. Note this yield is much lower than would be expected from a closed system built for producing biodiesel. Their costs are summarized in Table 5 and Table 6. Producing those 26.2 tons of algae biomass requires a capital investment of over three million dollars and a total annual cost of $933,995 – leading to a price of $35,649 per ton of algae biomass. There is a mistake in their setup which is corrected here.

**Table 5:** Capital costs of Grima closed system

Equipment	Cost (2003 dollars)
Photobioreactors x75	264,300
Centrifuge x2	247,898
Medium filter unit	18,014
Medium feed pumps x75	26,175
Medium preparation tank x3	104,442
Harvest broth storage tank x3	104,442
Centrifuge feed pumps x2	1682
Air compressors x3	78,309
Harvest biomass conveyer belts x2	14,200
Seawater pump station	13,661
Carbon dioxide supply station	3006
Weighing station	2366
Biomass silos x2	2740
	
Construction	Cost (2003 dollars)

Installation costs	264,371
Instrumentation and control	88,124
Piping	264,371
Electrical	88,124
Buildings	264,371
Yard improvements	88,124
Service facilities	176,247
Land	52,874
Engineering and supervision	220,309
Construction expenses	216,784
Contractor's fee	108,392
Contingency*	301,480
	
**Total capital cost**	3,014,803
**Annual cost**	429,240

*Mistake in original paper

**Table 6:** Yearly operating costs of Grima closed system

Input		Cost (2003 dollars)
Culture medium (at $0.5883/kg)	65,500 kg	38,534
Carbon dioxide (at $0.4706/kg)	96,940 kg	45,620
Media filters (at $70.59/unit)	210	14,824
Air filters (at $94.12/unit)	105	9883
Other consumables (at $117.65/kg)	13 kg	1529
Cooling water (included in pumping station)		0
Power (at $0.05883/kW h)	99,822k Wh	5873
Labor (at $16/h)	8760 hours	140,160
Supervision		28,032
Payroll charges		42,048
Maintenance		35,249
Operating supplies		442
General plant overheads		111,893
Tax		24,312
Contingency		5813
Wastewater treatment (at $0.59/m^3^)	10,480m^3^	6183
Capital costs (Table 5)		332,093
		
**Total yearly operating costs**		933,995
**Biomass costs ($/t)**		35,649

This is notably conservative in its estimations of non-input expenses. For instance, construction costs are ~70% of the total capital cost, while “general overhead”, about ~13% of total annual expenses, is fairly significant, along with labor taking ~17% of annual cost as well as various other miscellaneous costs that all add up. These are quite significant relative to the cost of consumable inputs (CO_2_, culture mediums, etc), which only make up ~13% of annual production cost. Construction costs, for one, can likely be reduced for later plants, similar to the cost savings that nuclear power plants encounter. In addition, although the algae farming systems that Molina Grima et al were studying were particularly complex, and it is possible that other bioreactor designs will be cheaper to put together. Finally, labor and general overhead can also be reduced when scaled past one plant, since a worker can then cover multiple plants.

Weissman and Goebel’s 1987 U.S. Department of Energy engineering report has their basic costs inflation adjusted to 2003. In addition, their CO_2_ price is greatly far out of line even after adjusting for inflation, since one of their central assumptions is cheap CO_2_. These are thus replaced with unit costs from the Molina Grima paper, which are close to current market prices, and recalculated. While other input prices have also fluctuated since 1987, they are not far off from current price quotations. In addition, electricity costs are equalized to the Molina Grima paper as well. The DOE open system specifies 192 hectares of ponds on a total of 384 hectares of land, producing 112 metric tons of biomass per hectare per year. Again assuming a 10% oil yield, this produces 11,200kg of oil/ha/year, or 3362 gallons/ha/year. Its cost per hectare is specified in Table 7 and Table 8. Capital costs are considerably lower for this system, totaling just over $100,000 per hectare, with a total annual cost of $147,769, or $1,319 per ton of algae biomass. The greatest expense for this system is the CO_2_ input, which makes up an impressive ~80% of the annual cost.

**Table 7:** Capital costs of DOE open system per hectare

Equipment	Cost/ha (2003 dollars)
Earthworks	16,613
Walls & structural	13,611
Mixing system	8,063
Carbonation system	3,000
Instrumentation	820
Primary (settling ponds)	12,259
Secondary (centrifuges)	6,488
Water supply/distrib.	7,255
C02 distribution	426
Nutrient supply	1,280
Salt disposal	1,365
Buildings (not for centf.)	939
Roads and drainage	854
Electrical distribution & supply	3,154
Machinery	684
Engineering/Construction/Contingency	19,203
Land (2 ha)	4,098
	
**Total capital cost**	101,256
**Annual cost**	14,417

**Table 8:** Operating costs of DOE open system per hectare

Inputs	Units/ha	Cost/ha (2003 dollars)
C02 (at $0.4706/kg)*	246,400 kg	115,956
N, as NH3 (at $0.25/kg)	5,936 kg	1,484
P, as Superphosphate (at $0.90/kg)	560 kg	504
Fe, as FeSO4 (at $0.50/kg)	560 kg	280
Flocculant (at $5/kg)	224 kg	1,120
		
Power (at $0.05883/kW h)		

Mixing	10,729 kWh	631
1 Harvesting	1,771 kWh	104
2 Harvesting	5,729 kWh	337
Water Supply	8,750 kWh	515
Nutrient Supply	521 kWh	31
Buildings	1,042 kWh	61
		
Other		

Salt Disposal (at $0.067/kg)	168,000 kg	11,256
Maintenance		511
Labor		562
Capital costs (Table 7)		14,417
		
**Total yearly operating costs**		147,769
**Biomass costs ($/t)**		1,319

*Cost per unit was $0.035/kg in original report

Unfortunately, there have been few public analyses of large scale industrial transesterification plants, despite the fact that several commercial biodiesel plants exist in various locations across the United States. Molina Grima 2003 does provide an analysis of a small-scale esterification process for both open and closed inputs. However, this paper was unclear about the specifics of the reaction despite detailed itemization, and furthermore is quite clearly designed as a small-scale operation. As such, we provide our own framework. A 1994 paper estimated that a large plant that can produce 30 million gallons of biodiesel annually, fueled by tallow, can be constructed for ~$15 million [15]. This ballpark number is supported by another analysis based off a soybean input [16]. This is surprising, since research into the actual capital costs of ethanol plants, which operate on a simpler reaction, revealed an average cost of $1.53 per gallon of capacity [17].

We present this analysis of the transesterification process economics in Table 9. Given the uncertainty of capital costs, we use a more conservative estimate, so our 30 million gallon plant will cost $46 million – equal to the cost of a similar sized ethanol plant. This is annualized to $6.55 million. Our inputs are methanol in a 6:1 molar ratio with oil input, NaOH in a 1:100 molar ratio with oil input, and hexane in a 1:1 volume ratio with methanol – a rather standard setup [12]. Production of 30 million gallons of biodiesel requires an input of 6.7 million to 25 million kilograms of oil by weight [15,16]. We assumed a requirement of 15 million kilograms. Requirements for other inputs are scaled from Molina Grima 2003. Finally, we assume an oil yield of 40% for a closed system and 15% for an open system. Capital costs are not a significant fraction of the operating cost, and the cost of biomass make up the bulk of the price of $45.12 or $4.99 per gallon for inputs from closed and open systems respectively. These are adjusted to $49.39 and $5.46 per gallon for energy equivalence to regular diesel.

**Table 9:** Costs of oil extraction

Variable inputs	Units	Cost ($)
Algal biomass, closed, ($35.64/t)^1^	37,500	1,336,824,488
Algal biomass, open, ($1.32/t) ^2^	100,000	131,936,202
Methanol (at $200/t)*	12,522	2,504,348
NaOH (at $500/t)**	26	13,043
Hexane (at $600/t)***	10,449	6,269,162
Cooling water ($0.0294/m^3^)	8,287,214	243,644
Steam (at $0.0049/kg)	56,250,000	275,625
Power (at $0.05883/kWh)	750,000	44,123
		
Fixed Inputs		

Labor(at $16/h)	12,000	192,000
Maintenance (at 10% annual capital costs)		654,937
Capital costs annualized		6,549,365
		
**Biodiesel cost, closed ($/gal)**		45.12
**Biodiesel cost, open ($/gal)**		4.99
**Biodiesel cost, closed, diesel equivalent ($/gal)**		49.39
**Biodiesel cost, open, diesel equivalent ($/gal)**		5.46

^1^15,000 tons of oil at 40% yield

^2^15,000 tons of oil at 15% yield

* http://www.icispricing.com/il_shared/Samples/SubPage135.asp

** http://www.icis.com/StaticPages/a-e.htm#C

*** http://www.icispricing.com/il_shared/Samples/SubPage90.asp

These numbers can be improved by relaxing some of the assumed costs. For closed system, the greatest cost of biodiesel production is in the capital outlay required to build out photo-bioreactors. Capital and fixed input costs are the most likely to be improved given with improved technology, experience, and economies of scale. On the other hand, the requirements for variable inputs such as CO_2_ are unlikely to be reduced without massive advances in algae engineering (which may yet happen); regardless, the variable inputs are not a major percentage of the total cost. In addition, photo bioreactors can likely reach much higher yields. Finally, hexane is used as a solvent in the transesterification reaction, and thus can be recycled for reuse. Sensitivity analysis for the closed systems is presented in Table 10. Given that the major cost of biodiesel is the cost of algae, and thus the capital cost of constructing photo bioreactor systems, it is difficult to imagine closed system sourced biodiesel being viable.

**Table 10:** Scenarios for closed system

	EE $/gal
1) Yield increased to 60%	33.13
2) Total capital + fixed costs of algae production reduced 50%	26.18
3) 60% yield, 50% capital/fixed cost reduction	17.65
4) 50% hexane recovery	49.28
5) 60% yield, 50% capital/fixed cost reduction, 50% hexane recovery	17.54

On the other hand, the major cost involved in biodiesel sourced from open pond systems is the cost of CO_2_ input, while the capital costs are very low. While yield can never be as high as in closed systems – 50% would appear to be a pipe dream for open ponds – they certainly can be improved. Finally, as before, hexane recovery could also reduce costs. Scenarios are presented in Table 11. Improved yields greatly help algae biofuels to nearly achieve the cusp of economic feasibility. However, it is somewhat difficult to envision improvements past 30% yields for open systems. Nevertheless, combined with reductions in CO_2_ costs, open pond sourced biodiesel is at the cusp of feasibility. Essentially, for algae to be close to economically feasible as a biofuel simply requires little to no CO_2_ cost and an open pond system with reasonable lipid yields. We will now go over possible policies that may tip the balance.

**Table 11:** Scenarios for open system

	EE $/gal
1) Yield increased to 20%	4.24
2) Yield increased to 30%	3.02
3) CO_2_ price of $0.2/kg (from $0.47/kg)	3.29
4) CO_2_ price of $0.035/kg (from $0.47/kg)	1.96
5) 50% Hexane recovery	5.34
6) 20% yield, $0.2/kg CO_2_ price	2.61
7) 30% yield, $0.2/kg CO_2_ price	1.94

### Current policy

The following section is an overview of the programs currently in effect in the United States that could incentivize the production of algae biodiesel. The incentives provided by these programs provide a template for the types of policies that could allow algae biodiesel to become profitable.

#### Renewable fuel standard

Administered by the Environmental Protection Agency, the Renewable Fuel Standard (RFS) was established by the Energy Policy Act of 2005 and was expanded upon in the Energy Independence and Security Act of 2007. The RFS is a provision that requires all transportation fuel to be blended with a certain amount renewable fuel, including bioethanol and biodiesel. Fuel producers were to blend 9 billion gallons of renewable fuel into the nation’s gasoline in 2008, with quotas increasing annually to 36 billion gallons in 2022. Notably, the expanded RFS mandates that an amount of this renewable fuel must be “advanced biofuels,” defined as biofuel produced with non-corn feedstocks that have at least 50% lower lifecycle greenhouse gas emissions than petroleum fuel. Of the 36 billion gallons mandated in 2022, for example, at least 21 billion gallons must be advanced biofuel. In terms of making algae more viable, the RFS does not directly incentivize its production. It does, however, guarantee a market for algae biodiesel, as it falls under the category of advanced biofuel [18].

#### Biodiesel tax credit

Biodiesel producers can claim a tax credit depending on the type of biodiesel produced. The credit is set at $1 per gallon produced of “agri-biodiesel,” which is defined as biodiesel produced from virgin agricultural products such as soybean oil or animal fats. Alternatively, producers of biodiesel from previously used agricultural products such as recycled fryer grease can claim a 50 cent per gallon tax credit [18]. Algae biodiesel would be likely to fall under the former category of agri-biodiesel, allowing it to claim the $1 per gallon tax credit. It should be noted that this tax credit is set to expire in December of 2009, but a bill to extend it for a further five years is currently under consideration [19].

#### Small Agri-Biodiesel Producer Credit

The Small Agri-Biodiesel Producer Credit is valued at 10 cents per gallon produced. It can only be claimed by a producer of agri-biodiesel with a production capacity of less than 60 million gallons of fuel per year, and can only be claimed on the first 15 million gallons produced in a given year. It is unlikely that algae biodiesel production facilities would produce at a low enough level to be considered “small,” but in the event that smaller production facilities are ideal, this tax credit could still be beneficial [18].

#### Biorefinery assistance

Introduced by the Food, Conservation, and Energy Act of 2008 and administered by the United States Department of Agriculture, the Biorefinery Assistance program offers loan guarantees and grants for the construction of biorefineries, facilities specializing in the creation of advanced biofuels. The program has received $75 million in mandatory funding for FY2009 and $245 million in FY2010 for loan guarantees. In addition, $150 million has been authorized annually for FY2009-FY2012 [18]. It is unclear how much of this funding would be available to the construction of a proposed open system facility for the production of algae biodiesel, but a grant is certainly possible.

#### Bioenergy program for advanced biofuels

Another program established by the Food, Conservation, and Energy Act of 2008, the Bioenergy Program for Advanced Biofuels provides payments to producers of advanced biofuels. The program has received annual funding through FY2012: $55 million for FY2009, $55 million for FY2010, $85 million for FY2011, and $105 million for FY 2012, with authorization for an additional $25 million each year from FY2009-FY2012 [18]. Again, it is not clear how much of this funding would go to an algae production facility, but at the very least, programs such as this show that the U.S. government is willing to devote large amounts of money to advanced biofuel programs. As research into algae biodiesel continues in the private sector, if it proves successful, it will be quite likely that government funding will be available to it in the future.

#### Import duty for fuel ethanol

This import duty is comprised of a 2.5% ad valorum tariff and a most-favored-nation duty of $0.54 per gallon of fuel ethanol imported into the United States from most countries. Ethanol imported from the Caribbean Basin Initiative countries may be exempt from these trade restrictions [18]. Like all import duties, it is unclear whether its effects on the world’s ethanol market are for the best, but its existence certainly shows that the United States government is interested in protecting the domestic biofuels market. It is likely that similar actions might be taken to protect domestic producers of algae biodiesel from foreign markets in the event that the method becomes a profitable way to produce biofuel.

## Conclusions

Our model clearly shows that closed system method of production of algae biodiesel, despite its immunity to contamination, is prohibitively expensive. The policies for incentivizing biofuel production that are currently in place, most notably the monetary assistance of the Biodiesel Tax Credit, could potentially allow algae biodiesel to be produced profitably using an open pond system given certain assumptions about the costs of algae biodiesel production (Table 12). In addition, the market created by the Renewable Fuel Standard offers profitability even if algae biodiesel does not meet these conditions.

**Table 12:** Scenarios with biodiesel tax credit

	EE $/gal
1) Closed system 60% yield, 50% capital costs, 50% hexane recovery	16.54
2) Open system, 15% yield	4.46
3) Open system, 20% yield	3.24
4) Open system, 30% yield	2.02
5) Open system, CO_2_ price of $0.2/kg (from $0.47/kg)	2.29
6) Open system, 20% yield, $0.2/kg CO_2_ price	1.61
7) Open system, 30% yield, $0.2/kg CO_2_ price	0.94

The most important of these conditions would be if the cost of CO_2_ becomes negligible or substantial increases in yield are observed. The simplest way of reducing CO_2_ costs would be through carbon trading. Since algae biodiesel is carbon neutral overall and consumes CO_2_ in the production process, it is in the prime position of being able to sell emissions credit. However, given current futures prices from the European Climate Exchange (Table 13), this method of offsetting CO_2_ costs is currently not feasible. Carbon trading schemes must become more robust, i.e. expensive, to allow an algae biodiesel producer to sell carbon offsets. Alternatively, the CO_2_ cost problem could be alleviated if the cost of commercial CO_2_ drops significantly. Increases in yield could result from advances in genetic engineering of algae so that they are better able to compete with contaminants, though this technology is currently far from implementation. Given the assumptions presented above, open-pond algae as a biodiesel fuel is close to feasibility as a full replacement for diesel, and currently can work well as a blend in petro diesel. Nevertheless, it cannot do so without subsidies, considerable technology improvements, or increases in the price of fuel. Thus the likeliest impacts on feasibility will depend on government policy towards carbon emissions and as always, future research.

**Table 13:** European climate exchange EUA futures (12/7/09)

Contract	Settlement price ($/ton)	Total volume
Dec09	21.87	7665
Dec10	22.24	9267
Dec11	23.16	1369
Dec12	24.27	1894
Dec13	25.86	50
Dec14	27.31	0

## Competing interests

The authors declare that they have no competing interests.

## Authors' contributions

YG provided technical insight and writing on the overall process, and helped with the numbers. CG provided research and writing on policy impacts. YL provided research on the costs involved and ran the numbers. DT & CT provided background research and drafted the initial manuscript. All authors contributed to writing the final manuscript.
